# The impact generated by publicly and charity-funded research in the United Kingdom: a systematic literature review

**DOI:** 10.1186/s12961-019-0425-2

**Published:** 2019-02-28

**Authors:** Daniela Gomes, Charitini Stavropoulou

**Affiliations:** School of Health Sciences, City, University of London, Northampton Square, EC1V 0HB London, UK

**Keywords:** Research impact, Public and charity funding, Evidence

## Abstract

**Objective:**

To identify, synthesise and critically assess the empirical evidence of the impact generated by publicly and charity-funded health research in the United Kingdom.

**Methods:**

We conducted a systematic literature review of the empirical evidence published in English in peer-reviewed journals between 2006 and 2017. Studies meeting the inclusion criteria were selected and their findings were analysed using the Payback Framework and categorised into five main dimensions, namely knowledge, benefits to future research and research use, benefits from informing policy and product development, health and health sector benefits, and broader economic benefits. We assessed the studies for risk of selection, reporting and funding bias.

**Results:**

Thirteen studies met the inclusion criteria. The majority of the studies (10 out of 13) assessed impact at multiple domains including the main five key themes of the Payback Framework. All of them showed a positive impact of funded research on outcomes. Of those studies, one (8%), six (46%) and six (46%) presented a low, moderate and high risk of bias, respectively.

**Conclusions:**

Empirical evidence on the impact of publicly and charity-funded research is still limited and subject to funding and selection bias. More work is needed to establish the causal effects of funded research on academic outcomes, policy, practice and the broader economy.

**Electronic supplementary material:**

The online version of this article (10.1186/s12961-019-0425-2) contains supplementary material, which is available to authorized users.

## Background

Every year, public and charity funding bodies in the United Kingdom invest significantly in health research. In 2014 alone, the United Kingdom Clinical Research Collaboration reported an expenditure of approximately £3.01 billion in health research funded by public and charity sources [1], with the Medical Research Council, the National Institute of Health Research (NIHR) and the Wellcome Trust being ranked among the top public and philanthropic funders of health research worldwide [2]. Thanks to this investment, a number of achievements have been possible and the United Kingdom “*has developed some of the strongest and most productive clinical medicine research bases in the world*” [3].

Given the magnitude of the investment in health research, there has been a growing interest in exploring the impact it generates. However, defining and measuring research impact is not straightforward; it is a complex exercise and the growing international interest on this subject has led to the development of multiple appraisal frameworks. A recent literature review identified 24 different methodological research impact frameworks that have been used in various countries and settings [4], with the Payback Framework being the most commonly used [5] both in the United Kingdom and internationally [4, 6, 7]. The Payback Framework was developed by Buxton and Hanney [8] and measures impact in five dimensions, namely knowledge, benefits to future research and research use, benefits from informing policy and product development, health and health sector benefits, and broader economic benefits.

Using the Payback Framework, this study’s aim is to review, synthesise and critically assess the empirical evidence of the impact generated by publicly and charity-funded health research in the United Kingdom. We do so acknowledging that there is inherent uncertainty regarding the outcomes of health research, in particular blue-sky and preclinical investigation [9]. We also accept that different types of research are expected to have different impact, with implications in the final assessment of research.

## Methods

### Data sources and searches

To address the study’s aim we conducted a systematic literature review. The Electronic Databases considered in this review were DoPHER (Database of Promoting Health Effectiveness Reviews), EBSCOhost CINAHL (Cumulative Index to Nursing and Allied Health Literature), EBSCOhost MEDLINE, OVID Embase, CENTRAL (Cochrane Central Register of Reviews of Effects), CDSR (Cochrane Database of Systematic Reviews), DARE (Database of Abstracts of Reviews of Effects), and NHS EED (NHS Economic Evaluation Database). Searches were conducted using a combination of keywords and index terms (for example, MeSH terms) combined with Boolean operators, as part of an extensive search. These were adapted to the other relevant electronic databases using the synonyms used by each database. The search terms included combinations of different relevant terms, such as ‘budget’, ‘funding’, ‘investment’, ‘grant’, ‘research support’, ‘clinical research’, ‘health research’, ‘medical research’, and ‘impact’, ‘value’, or ‘research outcome’. A full list of search terms and an example of the search strategy of the three main databases (OVID Embase, EBSCOhost CINAHL and EBSCOhost MEDLINE) is available in Additional file 1 to enable replication. One author (DG) performed the search of the electronic databases.

In addition, citation tracking of relevant literature and reference lists of the identified studies and reports were searched for further studies by both authors. Hand searches were also conducted in the key journals *Value in Health*, *The Journal of Health Economics* and *BMC Health Services Research*.

### Study selection

We included empirical studies that (1) explicitly analysed the impact generated by research funding provided by public and charity bodies in the United Kingdom, (2) analysed the impact generated in the United Kingdom or other countries, (3) were published between 2006 (the year the NIHR was lunched) and 2017, (4) were peer reviewed and (5) were written in English. We excluded speculative studies, opinion papers or editorials that discussed the potential impact generated by funded research in the United Kingdom and studies that did not focus on health-related research.

The selection of the relevant studies followed the steps identified by the PRISMA guidelines for systematic reviews and meta-analyses [10]. First, we screened all records on the basis of title and abstract. When it was not possible to determine whether a study met the inclusion/exclusion criteria based on title and abstract review, we included the study for full text review. Both authors screened all records, assessed the list of studies included for full text review and agreed on the final list.

### Data extraction and data synthesis

Following the identification of the studies, data extraction and quality assessment was conducted using a standardised data extraction form. Extracted data included aims, programme assessed, methods, outcomes assessed and main findings. Given the diversity of the methodological approaches used in the identified studies, a quantitative analysis of the results was not possible. Overall, the results were synthesised into the five key domains of the Payback Framework, using the adaptation discussed in Donovan and Hanney [11], in which payback from research includes the following five main dimensions and definitions:

1. Knowledge, including articles published in peer-reviewed journals, conference presentations, books, book chapters and research reports.
2. Benefits to future research and research use, such as better targeting of future research, development of research skills, personnel and overall research capacity, a critical capacity to appropriately absorb and utilise existing research, including that from overseas, staff development, and educational benefits.
3. Benefits from informing policy and product development, improved information bases for political and executive decisions, other political benefits from undertaking research, and development of pharmaceutical products and therapeutic techniques.
4. Health and health sector benefits, including not only health improvements but also cost reduction in delivery of existing services, qualitative improvements in the process of delivery, and improved equity in service delivery.
5. Broader economic benefits, wider economic benefits from commercial exploitation of innovations arising from research and development (R&D), and economic benefits from a healthy workforce and reduction in working days lost.

### Risk of bias assessment

Given the heterogeneity of the studies included, we decided to develop a simple tool to assess the risk of bias. The tool focused on three main types of risk of bias, namely funding, selection and reporting bias. Funding bias refers to the potential tendency for the study to support the interests of the sponsor. Selection bias occurs when selected samples (including individuals, groups or data) are not representative of the population being reviewed. Reporting bias refers to the reported data favouring certain outcomes or key aspects of the study not being clearly described.

Each study was assessed and was given a score for each of the three types of bias using the risk of bias tool described in Table 1. An overall score was then calculated in the following way – studies with no high-risk score in any of the three domains were classified as ‘low risk of bias’, those with two high-risk scores were classified as ‘moderate risk of bias’ and those with three high-risk scores were classified as ‘high risk of bias’.

**Table 1:** Risk of bias assessment tool

Risk of bias	Rationale	Relevant points and relative scoring (1 = low risk; 2 = medium risk; 3 = high risk)^a^
Funding bias	Potential tendency for the study to support the interests of the sponsor	What was the role of the evaluated funding body in the financing of the study? 1. The study or its author(s) did not receive funding from the body they evaluate 2. The study or its author(s) were partly funded by the body they evaluate 3. The study or author(s) was funded exclusively by the body they evaluate
Selection bias	The selected samples (including individuals, groups or data) are not representative of the population studied	Is the selected sample likely to be representative of the population being reviewed? 1. Very likely 2. Somewhat likely 3. Not likely
Reporting bias	The reported outcomes are inaccurate or inappropriate	Are all aspects of the study (including aims, methods and results) clearly described and reported? Is there a balance between reported data/opinions and critical analysis or are these consistently more favourable of the outcome/funding in analysis? 1. All aspects of the study clearly described with supporting evidence 2. Some aspects/evidence of the study are not clear 3. Several aspects/evidence of the study are missing with difficult interpretation of the findings

^a^Overall rating for risk of bias: 1 (strong; no high-risk score); 2 (moderate: 1–2 high-risk scores); 3 (weak; 3 high-risk scores)

## Results

### Study inclusion

**Fig. 1:**
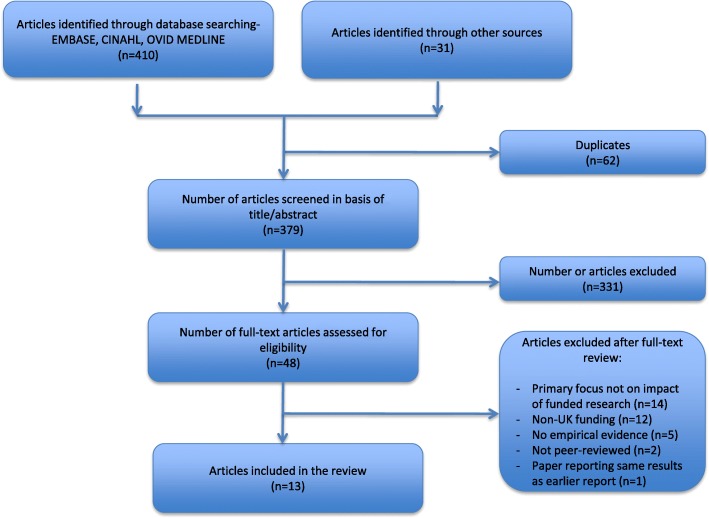
Flow diagram of study selection following the PRISMA guidelines

The process of study selection is summarised in Fig. 1 and follows the PRISMA guidelines. From the main database searches (CINAHL *n* = 94, EMBASE *n* = 204 and MEDLINE *n* = 112) a total of 410 studies were identified. An additional 31 studies were identified by other searches, including CENTRAL, CDSR, DARE, DoPHER, Google Scholar, National Institute for Health and Clinical Excellence (NICE) and NHS EED, as well as key journals, citation tracking and the main funding websites. After removing 62 duplicates, we proceeded with the review of the title and abstract of the remaining 379 studies. At this stage, 331 studies were excluded and the remaining 48 were retrieved as full text for further review, which are listed in Additional file 2.

Following full text review, 34 studies were excluded; 14 did not have a primary focus on the impact of public and charity funded research, 12 considered funding outside the United Kingdom, five did not provide empirical evidence, two were not peer-reviewed and one was published before 2006. One study was excluded to avoid duplication since it was a brief journal article [12] of an earlier report [13]. This resulted in 13 studies being included for analysis.

### Study characteristics

The data extraction table can be found in Additional file 3. The funding body mostly assessed in the studies included was the NIHR. Three studies focused on the NIHR’s Health Technology Assessment (HTA) Programme, one on its Service Delivery and Organisation (SDO) R&D Programme, one study looked at the NIHR’s Biomedical Research Centre at Oxford, one explored the impact of two NIHR-funded research networks, one analysed the NIHR-funded Cochrane Review Groups (CRGs) and, finally, one looked at the 10 years of the NIHR. Four studies looked at specialised charities including the United Kingdom Occupational Therapy Research Foundation, Asthma UK, the National Cancer Research Institute and leading funders of cancer research in the UK. Finally, one study took a wider approach looking at the general economic effect of government and charity research expenditure on pharmaceutical R&D.

Out of the 13 studies, seven (54%) used mixed methods, three (23%) used quantitative and three (23%) used qualitative methods. A number of different designs were used, including questionnaire surveys (*n* = 4), qualitative interviews (*n* = 5), case studies (*n* = 3), document review (*n* = 3), bibliometric analysis (*n* = 3) and quantitative analysis using econometric techniques (*n* = 3). The design of the studies was such that none of the studies could establish causality between research funding and various outcomes. Only one study explored the hypothesis that publicly and charity-funded research led to increased pharmaceutical R&D, but using appropriate econometric tests, it showed that there may be a dual causal relationship between the two. The authors therefore conclude that their results should be interpreted as a positive association between public expenditure and private R&D, and cannot claim causality [14].

### Risk of bias

All reviewed studies had received funding, partly or exclusively, by the organisation they were assessing. High risk of selection bias was present in seven out of the 13 studies (54%), as they chose to analyse funded projects that were more likely to have shown positive impact. Finally, reporting bias was high in more than half of the studies (8/13; 62%), either because important information regarding the methods, funding sources or results was missing, or because the overall claims of the studies highlighted predominantly data showing positive impact of research. Overall, from the included studies only one presented a low risk of bias (8%). All of the remaining included studies presented a moderate (6/13; 46%) to high risk of bias (6/13; 46%), accounting for an estimated 92% of the included studies. The results are presented in Table 2.

**Table 2:** Risk of bias assessment

Author/Year	Funding bias	Selection bias	Reporting bias	Total score
Bunn et al., 2014 [15]	3	2	2	2
Glover et al., 2014 [24]	2	3	2	2
Guthrie et al., 2015 [16]	3	2	2	2
Guthrie et al., 2016 [23]	3	3	3	3
Hanney et al., 2007 [13]	3	1	2	2
Hanney et al., 2013 [20]	3	3	3	3
Lichten et al., 2017 [25]	3	3	3	3
McCrae et al., 2012 [22]	3	2	3	2
Morgan Jones et al., 2016 [21]	3	3	3	3
Peckham et al., 2008 [19]	3	3	3	3
Sainty, 2013 [17]	3	3	3	3
Sullivan et al., 2011 [18]	2	1	3	2
Sussex et al., 2016 [14]	2	2	1	1

### Data analysis

The results of the final 13 studies were synthesised in accordance to the Payback Framework domains.

#### Knowledge

Eight of the included studies report research impact as a form of knowledge generation [13, 15–21]. Two of these studies focused on the NIHR’s HTA Programme. In 2007, Hanney et al. [13] assessed the impact of the first 10 years of the programme and showed that the mean number of publications per project was 2.93 (1.98 excluding the monographs). A more recent study by Guthrie et al. [16] reported that an estimated 96% of the NIHR-funded HTA Programme studies are published in the journal *Health Technology Assessment*; the authors claimed that work funded by the HTA Programme is cited more than twice as frequently as would be expected on average.

Two more studies looked at knowledge generated by programmes funded by the Department of Health in the United Kingdom. Peckham et al. [19] assessed the impact of the first 5 years of the SDO Programme (2001–2006) and showed that, of the 23 research projects, a total of 39 papers had been published in peer-reviewed journals by early 2006, equivalent to 1.7 articles per project. In addition, there were 95 national and international conference presentations. Each of the 23 research projects produced an average of 6.7 citations. Another study by Bunn et al. [15] assessed the reviews published by 20 NIHR-funded CRGs, showing that 1502 out of 3187 (47%) new and updated reviews published on the Cochrane Database between 2007 and 2011 were published by the 20 CRGs. In addition, in a sample of 60 reviews (out of 1502), 27 presented 100 or more citations and five out of 60 were cited over 400 times in Google Scholar.

Two studies looked at knowledge generated by two charity funders. Hanney et al. [20] described the various impacts identified from a range of Asthma UK research by conducting a survey among investigators of funded projects. Of the 90 projects that were returned, they showed an average of four peer-reviewed journal articles per project. Four respondents did not record producing any articles. As the authors pointed out, the results may be biased as it is possible that those projects for which surveys were not returned had a much lower number of publications. Another study by Sainty [17] looked at the United Kingdom Occupational Therapy Research Foundation (UKOTRF) by sending a questionnaire form to 11 grant holders. They concluded that knowledge generation was the main self-reported outcome by previous grant holders; this outcome was measurable through reported scientific publications in peer-reviewed journals (publications *n* = 6; submissions *n* = 14), publication of books/book chapters (*n* = 2), Cochrane review citations (co-authorship of a Cochrane review *n* = 1) and conference presentations (presentation at UKOTRF *n* = 1; submission to annual conference and other national and international conferences *n* = 39). They note that it is a requirement of the UKOTRF grant holders to submit an article to the *British Journal of Occupational Therapy* and an abstract to the organisation’s annual conference.

Sullivan et al. [18] compared the period before and after the launch of the National Cancer Research Institute in the United Kingdom in 2001, an initiative that brought together charity and public funders in the area of cancer research. They showed that UK cancer centres published just over one-eighth of all UK outputs (papers per year) in 1995 but almost a quarter by 2004.

Finally, Morgan Jones et al. [21] highlighted the impact of the NIHR in managing and sharing knowledge resources via the Journals Library and BioResource.

#### Benefits to future research/research use

Better targeting of future research was reported as an outcome of funded research in four of the reviewed studies [13, 15, 16, 21]. In particular, the study by Morgan Jones et al. [21] that reviewed a number of NIHR cases gave a lot of emphasis on better targeting of future research as a result of NIHR-funded research. The report presented 10 cases showing collaboration with charities and the third sector, including, for example, the collaboration of NIHR with the Stroke Association in research that has resulted in earlier and more efficient diagnosis for stroke survivors with cognitive impairment. In their analysis of the NIHR-funded CRGs, Bunn et al. [15] showed that 13 out of 60 Cochrane reviews conducted by the CRGs were cited in protocols or primary research. In addition, respondents of the survey provided 40 examples where they felt their reviews influenced primary research. However, Bunn et al. [15] reported that most of these examples of impact relate to work conducted by the Cochrane reviewers themselves. Therefore, there is limited evidence of a broader impact.

Two studies analysing NIHR’s HTA showed the programme’s contribution to future research. Hanney et al. [13] showed that 61 (46%) HTA projects went on to receive further funding, yet it is not clear whether this funding was from the HTA or other bodies. A few years later, Guthrie et al. [16] showed that HTA funding contributed to the development of new research methods, by stimulating their research field more widely or by introducing new research priorities as a result of its findings. This evidence was provided in approximately 58% of the case studies, with examples of the studies provided as supporting evidence. Half of the studies were extended; however, there was little evidence that there was broader impact beyond those already holding HTA grants.

Six of the reviewed studies provided a series of examples through which funded research has contributed to the development of research skills, personnel and overall research capacity as well as enabling staff development and educational benefits [13, 16, 18, 19, 21, 22]. Guthrie et al. [16] addressed the HTA Programme’s contributions to career development of researchers and overall research capacity through interviews of 20 stakeholders. Six out of the 20 interviewees reported contributions to career development and overall research capacity. The authors indicate that capacity-building is hard to establish, as most of the researchers receiving the grant were already established. In an earlier study, Hanney et al. [13] showed that 28 projects out of 133 completed questionnaires reported that qualifications had been gained or were expected to be gained from involvement in HTA projects. In addition, eight out of the 16 case studies they analysed reported that researchers involved in the funded projects progressed in their career through promotions, though the authors acknowledge that it is hard to tell this was the outcome of the funded project.

The report by Morgan Jones et al. [21] gave particular emphasis on the development of overall research capacity by developing or facilitating knowledge improvement and research teams in areas of research otherwise difficult to address. This was reported in three studies, namely (1) the Radiotherapy Trial Quality Assurance Team, supporting medical staff implementing research knowledge to practice; (2) the Hyper-acute Stroke Research Centres, enabling patient-informed decision and participation in treatment therapy trials to improve the delivery of better emergency care; and (3) the Enabling Research in Care Homes, involving the development of a research network at care homes. According to Morgan Jones et al. [21] the NIHR also recognises the relevance of retaining researchers following completion of funded research. The report referred to the Doctoral Research and Clinical Research Fellowships, the Leadership Programme and the Mentorship for Health Research Scheme. Furthermore, the report provided evidence that NIHR funding has contributed to the critical capacity to absorb and appropriately utilise existing research, including international research in five studies. The evidence was reflected in the centralisation of specialist cleft services and support in national and international trials; standardisation of children eczema treatment based on international guidelines; improvement of rehabilitation programme to stroke survivors; introduction of a personalised care approach to elderly patients with dementia; and collaboration in testing of artificial knee joints produced by foreign manufacturers.

McCrae et al. [22] examined the impact of two NIHR-funded research networks on a multi-centre randomised controlled trial of antidepressants in people with depression superimposed on dementia. The two networks are the Mental Health Research Network and the Dementia and Neurodegenerative Diseases Research Network. They showed that the Mental Health Research Network helped in gaining local ethics committee and NHS trust approvals, which can be a time-consuming process. Clinical study officers boosted a recruitment campaign and contributed to the monitoring and assessment of participants. The study mentioned a number of limitations of the networks, including potential problems of duplication or unclear roles and responsibilities, a degree of unrealistic expectation from principal investigators and additional bureaucratic burden.

The study by Sullivan et al. [18] showed that, following the establishment of the National Cancer Research Institute, there has been an increase in United Kingdom cancer centre collaborations with European (5–28% of all their outputs) and United States (6–21%) investigators. Peckham et al. [19], evaluating the first years of the SDO, argued that the projects demonstrate contributions to building the capacity of the workforce, as there are many examples from the bibliographic analysis and the case studies where the knowledge is used in teaching in universities. There was also some evidence that the research is stimulating user involvement in research.

In charity funding, Hanney et al. [20] showed that at least 62 higher degrees have been obtained or were expected, at least partly, as a result of Asthma UK’s project funding. In addition, 64% of the funded projects participating in the survey reported some career development, including promotion for principal investigators, further fellowships from major funders and recognition in the asthma field as a result of the Asthma UK project funding. Sainty [17] reported that funded research by the UKOTRF has not only contributed to promotion of the researcher’s profile and career progression but also to overall research capacity by enabling collaborative working with clinical organisations, universities and charitable partners. In addition, funding led to employer and other partner/host organisations contributions and attracted follow-on funding from external sources.

#### Benefits from informing policy and product development

Eight of the included studies assess the benefits of informing policy as a form of research impact [12, 13, 15–17, 19, 20, 23]. The main methods used to measure the impact were self-reported evidence from recipients of grants, citations in policy documents and case studies.

Surveys were used in four studies [13, 16, 17, 20]. Three out of the 11 respondents in the study by Sainty [17] reported that their findings were particularly relevant to inform discussions at a national policy event (*n* = 1) and national and regional dissemination forums (*n* = 2). Using a questionnaire survey, Hanney et al. [13] showed that 73% of the respondents claimed their study had an impact on policy to date, particularly for NICE projects. Hanney et al. [20], looking at Asthma UK, showed that 13% of the respondents claimed to have made an impact on policy, and 17% expected to do so in the future. Using Researchfish data, Guthrie et al. [16] provided evidence that 15% of the portfolio of studies analysed had an impact on policy, including participation in advisory committees and citations in clinical reviews, policy documents and guidelines.

Citations in policy documents were used as evidence of policy influence in two studies [15, 18]. Bunn et al. [15] claimed that Cochrane reviews have contributed to informing policy, with an estimated 722 citations identified in 248 guidelines and 481 reviews cited at least once. They included several sets of developed guidance at a local (*n* = 10), national (*n* = 175) and international level (*n* = 62). Sullivan et al. [18] showed that, after the establishment of the National Cancer Research Institute in the United Kingdom, there has been an increase in the number of citations on clinical guidelines and the press (BBC).

Interestingly, the evaluation of the SDO by Peckham et al. [19] reveals differences in evidence of policy influence depending on the method used. The literature review they conducted did not identify any citations in the documents related to policy, but evidence from interviewees indicates other informal mechanisms in which the knowledge was transmitted to policy such as meetings with the Department of Health. This demonstrates that knowledge can be effectively transferred in different ways but these may be difficult to trace when building an understanding of knowledge flow and research output. It has been difficult to confirm the use of SDO-funded research by practitioners through case studies. The data on outputs show that there is potential for a wide range of practitioners to access the research.

Certain studies referred to specific policies or policy organisations mainly due to the nature and scope of the funding body assessed. Guthrie et al. [16] showed that funding by the HTA Programme enhanced the existent collaboration between the programme, NICE and the National Screening Committee, who represent powerful identities in the process of disseminating research findings through policy and guidelines. According to Guthrie et al. [16], 15% of the overall portfolio of HTA-funded studies reported having some impact on policy. HTA Programme research has the potential to constitute and improve the information base for policy development and redesign at both national and international levels; however, it is not primarily involved in the direct process of policy development. Guthrie et al. [23] presented similar findings in a later study. Earlier assessments of the HTA showed similar impacts. Hanney et al. [13] showed that the HTA’s Technology Assessment Reports for NICE had the clearest impact on policy in the form of NICE guidance. Other bodies where the projects had impact included the National Screening Committee, the National Service Frameworks, professional bodies or the Department of Health. The case studies they presented provided considerable detail about the exact names of the policy documents informed by specific HTA projects, and the precise issues in the documents that were influenced by the specific research.

Finally, evidence that publicly funded research leads to development of pharmaceutical products and therapeutic techniques was found in one of the reviewed studies only. Morgan Jones et al. [21] provide evidence on the development of therapeutic techniques which are “*safer, less invasive and more focused on patients’ quality of life*” by NIHR-funded research. They also provide evidence from seven funded studies, which led to more efficient and cost-effective treatments.

#### Health sector benefits

Regarding improved health outcomes, the majority of the studies provided self-reported evidence. In the study by Sainty [17], three out of 8 respondents reported that their research findings were being applied to local practice, yet no specific details are given. Respondents to the study by Bunn et al. [15], assessing the impact of NIHR-funded Cochrane reviews, reported that 19 out of 60 of the Cochrane reviews assessed indicated potential to lead to health and health sector benefits. However, the majority were unable to provide evidence if these have led to changes in practice, improved health, improved equity and quality in service deliver, or cost reduction in delivery of existing services. According to Hanney et al. [20], only a small minority (10%) of the respondents sponsored by Asthma UK claim to have already made an impact in any of the various forms this could take, with 6% believing they had made an impact specifically to health. In addition to the survey, three of the 14 case studies analysed by Hanney et al. [20] “*described health gains from Asthma’s UK-funded contributions to research on leukotriene receptor antagonists and on immunotherapy for allergic rhinitis, and the potential health gains from research on peptide immunotherapy*”. Case studies were also used by Peckham et al. [19] to demonstrate a range of ways in which NHS managers and policy-makers have used SDO-funded research to develop service delivery.

Morgan Jones et al. [21] provide more specific evidence on research findings that have contributed to improved health and improved equity in service delivery nationally and internationally. The evidence in the three studies provided include a reduction of people dying from traumatic injury bleed by administration of tranexamic acid, reduction of post-operative complications by using WHO’s Surgical Safety Checklist and a reduction of the number of people at risk of death by withdrawing co-proxamol.

Two studies, whose primary focus was to estimate the economic benefits of funded research, provided evidence of health gains. Glover et al. [24], analysing the economic returns from United Kingdom publicly and charity-funded cancer-related research, estimated that there were 5.9 million QALYs gained from the prioritised interventions from 1991 to 2010. Similarly, Guthrie et al. [23] estimate economic benefits as monetary gains over QALYs. However, a total number of QALYs gained is not provided.

There was one study that showed mixed findings. Lichten et al. [25] evaluated the impact of the Oxford Biomedical Research Centre, which is funded by the NIHR. They interviewed both research leaders and senior clinicians, and founded interesting differences between the two groups. The research leaders identified a wide range of beneficial impacts that they expected might be felt at local hospitals as a result of their research activity. The senior clinicians responsible for patient care at those hospitals presented a more mixed picture, identifying many positive impacts, but also a smaller number of negative impacts, from research activity, such as duplication of roles.

Four studies reported qualitative improvements in the process of delivery and cost reduction in delivery of existing services. The study by Sainty [17] suggested that the funded studies assessed have contributed to quality improvements and cost reduction in delivery of existing services. One respondent to the survey reported that work was already being carried out to translate the research findings into commissioning of services by the local trust, which is likely to contribute to more cost-effective service delivery. Another respondent claimed that the implementation of the research findings to clinical practice had the potential to lead to safer and more cost-effective services.

Morgan Jones et al. [21] also drew attention to the contributions of NIHR-funded research to quality improvements in the process of delivery and cost reduction in delivery of services in a diverse range of areas, including improvement to screening methods in newborn babies, improvement to stroke prevention and reduction of associated costs, implementation of new therapies by reduced cost of antipsychotic medication in dementia patients by implementation of cognitive stimulation therapy, improved health promotion, improved commissioning of services, and prevention of spreading of life-threatening communicable diseases.

According to the interviews they conducted, Guthrie et al. [16] showed that the HTA Programme is recognised as high-quality funded research and, as a result, more likely to be translated into clinical guidelines, applied to practice and ultimately lead to improved health. Case studies were also analysed and seven out of 12 studies provided evidence that the HTA Programme has contributed to health and health sector benefits, which included improved health, improved equity in service delivery and more cost-effective service delivery. Despite this, is it not possible to infer that health benefits resulted directly as a unique result of the HTA Programme. Some of the studies analysed failed to provide significant evidence on this matter, with the possibility that the final outcome has resulted from a combination of factors, including multiple studies’ findings applied to practice. Additionally, two of the studies concluded that their findings matched the current guidance already in place, limiting the interpretation of the value generated by their research.

Similar challenges were seen in the study by Hanney et al. [13]. Eight out of the 16 studies reported it was impossible, unlikely or unrealistic to show any health gains, or that the evidence was too limited to show any health improvements. Another six talked about potential health gains and two discussed health improvements.

#### Broader economic benefits

Six of the included studies assess the economic impact as a result of research findings. Glover et al. [24] assessed the monetary returns from publicly and charity-funded cancer-related research. They showed that, in 2011/2012 prices, the net monetary benefit of the 5.9 million QALYs gained from the prioritised interventions from 1991 to 2010 was £124 billion. Their estimated internal rate of return incorporated an estimated elapsed time of 15 years. The paper related 17% of the annual net monetary benefit estimated to be attributable to United Kingdom research (for each of the 20 years between 1991 and 2010) to 20 years of research investment 15 years earlier (that is, for 1976 to 1995); this produced a best-estimate internal rate of return of 10%.

Sussex et al. [14] inferred that there are wider economic benefits from public and charitable R&D expenditure, as well as a correlation between this and private R&D expenditure; every £1 of public research expenditure is associated with an additional £0.83–£1.07 of private sector R&D spend. They show that 44% of that supplementary private sector expenditure occurs within 1 year, with the remainder accumulating over decades. This spill-over effect implies a real annual rate of return (in terms of economic impact) to public biomedical and health research in the United Kingdom of 15–18% and, when combined with previous estimates of the health gain that results from public medical research in cancer and cardiovascular diseases, the total rate of return would be approximately 24–28%.

Morgan Jones et al. [21] provided evidence from seven studies on the wider economic benefits from commercial exploitation of innovations arising from R&D, through NIHR-funded research at the levels of attraction of private funding for public–private partnerships to further potential, investment in innovative research that leads to development of pioneering advanced prototypes, investment in quantifying impacts of new therapies that can improve patients’ lives whilst reducing current expenditure, and attracting foreign manufactures investment in the United Kingdom for trial of innovative drugs, devices and diagnostics.

Guthrie et al. [16], in their overall assessment of the HTA Programme, claimed that there is little overlap between HTA and industry and showed that half of the studies showed impact on industry. The team then went ahead to conduct a full economic analysis of the impact of the HTA Programme [23], finding significant economic impact and arguing that, if 12% of the potential net benefit of implementing the findings of this sample of 10 studies for 1 year was realised, it would cover the cost of the HTA Programme from 1993 to 2012.

Yet, the evidence was not always easy to show. Hanney et al. [20] provided limited evidence that Asthma UK-funded research brought benefits to the broader economy, though they do mention the development of two spin-out companies in the United Kingdom resulting from this. The study by Sainty [17] stated that only one out of eight respondents addressed economic impact by claiming that the submission of an economic paper to the *British Journal of Occupational Therapy* had the potential to generate economic benefits. Yet, there is no clear evidence on how this economic benefit would be generated and to whom or which services.

## Conclusions

Funding is absolutely vital for research and funders and researchers are under pressure to show that money is well spent. Our study shows that the number of peer-reviewed papers that explore the impact of publicly and charity-funded research in the United Kingdom is limited, but it is growing. The majority of the studies reviewed (10/13) assessed impact at multiple domains of the Payback Framework. All studies argue that publicly and charity-funded research has a positive impact on most domains. Impact on knowledge was the easier dimension to quantify and measure, using bibliometric techniques such as number of publications and citations generated per project. In other domains, including impact on policy and practice, it is harder to demonstrate impact and evidence is mainly self-reported. However, it is expected that the Research Excellence Framework, a performance-based research funding system of higher education institutions in the United Kingdom [26], is likely to improve the way impact outside academia is assessed and measured and force researchers to devise better tools to achieve this. Yet, care should be given to avoid the selection and reporting bias that current Research Excellence Framework cases are likely to present when reporting impact.

The majority of the studies presented moderate to high risk of bias. All studies had received funding from the body they were assessing, raising concerns about the potential tendency of these studies, whether real or perceived, to support the interest of the funder. This is a concern raised by the authors of some of these studies [26], calling for more independent funding streams for research evaluation. In addition to funding bias and perhaps not unrelated to it, eight out of 13 studies showed high risk of reporting bias, highlighting the aspects or domains that were more likely to show high impact than those that did not. This may well reflect that certain domains are harder to measure, and should not undermine the fact that some of these studies put a significant amount of resources and effort in analysing cases, interviews and bibliometric databases and evidence of triangulation of different methodologies [13, 16, 21]. Finally, seven out of 13 studies had a high risk of selection bias, focusing purposively on cases, which were likely to create impact, therefore tending to overestimate the general impact of funded research. The use of purposive sampling may well be considered the best way of providing evidence of funded research “*which has generated benefits to and wider impacts on health*” [21] and the authors were very transparent in stating that in their reports. As Morgan Jones et al. clearly put it, their “*study was commissioned as a synthesis of impacts and benefits, not an evaluation*” [21] and acknowledge this as a limitation of their study.

Our review is not without limitations. We included in our analysis only peer-reviewed studies to ensure that the studies had been subject to criticism and met the standards for scientific publication. This means that we excluded studies that may be reporting on the impact of funded research but were not peer reviewed. Indeed, some funders themselves put significant effort to celebrate their successes, including, for instance, the Medical Research Council’s annual impact reports [27] and the Wellcome Trust’s system of tracking the career of its fellows [28]. We also acknowledge the challenge of extracting precise information from all studies, given the heterogeneity and nature of some of them. As an example, the study by Morgan Jones et al. [21] included 100 case studies showing impact on different aspects of the Payback Framework, which made the summary and synthesis of results difficult. In addition, we chose to look at the literature from 2006 onwards, as the launch of the NIHR that year was seen as a significant change in public funding of research in the United Kingdom. This choice means that we may have missed out on impact assessment studies that were conducted before 2006. Finally, we acknowledge that our choice of journals to hand-search was likely to be restricted and a wider range of journals could have been added.

Our results could be of potential interest to public and charity funders. The limited number of studies identified in this review highlights the need for a more systematic collection of data that will enable them to show that their investment offers value for money, in particular in areas where evidence is harder to establish such as policy development and wider societal effects. Significant effort towards that direction has been made in the last decade, including the introduction of Researchfish^®^ [29], a comprehensive digital platform to collect research outputs and other elements of wider impact produced by each research project funded by a large number of United Kingdom research organisations. These platforms are not without caveats, as they rely on self-reporting of outcomes, but they have certainly helped to embed the idea that at the end of a project impact should be both measured and reported. Other funders have developed spreadsheets to allow researchers to report specific outputs arising from research projects supported by the funder [28].

There is recognition that research activities have a degree of risk and unpredictability [30] and that scientific knowledge is often obtained in the process of trial and error. As a result, thorough assessment of health research impact can be encountered with some criticism and interpreted as “*neglecting the inherent value of science*” [31]. Nevertheless, some degree of evaluation of funded research is needed to ensure transparency and accountability as research funding entities are subject to growing pressure to demonstrate the impact generated by their funded research. Our study suggests that there is still space for improvement.

## Additional files

Additional file 1: Main databases search strategies. (DOCX 97 kb)

Additional file 2: Full papers for review. (XLSX 12 kb)

Additional file 3: Data extraction table. (DOCX 29 kb)

## Abbreviations

CINAHL: Cumulative Index to Nursing and Allied Health Literature
CRG: Cochrane Review Groups
HTA: Health Technology Assessment
NHS: National Health Services
NICE: National Institute for Health and Clinical Excellence
NIHR: National Institute for Health Research
R&D: research and development
SDO: Service Delivery and Organisation
UKOTRF: United Kingdom Occupational Therapy Research Foundation
