# Supplementary material for: The impact generated by publicly and charity-funded research in the United Kingdom: a systematic literature review
Source: Health Res Policy Syst. 2019 Feb 28;17:22. doi: 10.1186/s12961-019-0425-2 (PMC6394081; doi:10.1186/s12961-019-0425-2)
Supplement: Supplementary file 1 — Main databases search strategies. (DOCX 97 kb) [file 12961_2019_425_MOESM1_ESM.docx]

## **Additional file 1**: Main Databases Search Strategies

The following tables represent the search strategies used for the three main dabatase searches – OVID EMBASE, EBSCOhost Medline®, and EBSCOhost CINAHL®. The results presented on the table reflect a search last re-run on the 2^nd^ of February 2018.

The terms on the table were combined within columns with “OR” and between columns with “AND”.

OVID EMBASE - SEARCH STRATEGY

| **#** | **Searches** | **Results** |
| --- | --- | --- |
| **1** | *budget/ or *funding/ or *investment/ | 9502 |
| **2** | (budget* or grant* or fellowship* or scholarship* or research support).ab | 64107 |
| **3** | 1 or 2 | 71414 |
| **4** | clinical research/ or medical research/ | 251356 |
| **5** | health research.ab. | 10937 |
| **6** | 4 or 5 | 258233 |
| **7** | (research outcom* or research impact or research value or economic impact or contribution*).ab. | 299491 |
| **8** | 3 and 6 and 7 | 254 |
| **9** | limit 8 to (abstracts and english language and english and yr="2006 - 2017") | 204 |

EBSCOhost MEDLINE® SEARCH STRATEGY

| **#** | **Searches** | **Limiters/Expanders** | **Results** |
| --- | --- | --- | --- |
| **S1** | (MH “Budgets") OR (MH "Health Services Purchasing")OR (MH "Financial Support") OR (MH "Investments") OR (MH "Resource Allocation") OR (MH "Health Resource Allocation") OR (MH "Research Support") OR (MH "Training Support, Financial") | **Limiters** – Abstract Available; Published Date: 20060101-20171231; English Language; Human. **Search modes** – Find any of my search terms | 3,562 |
| **S2** | "scholarship" OR "funding" OR "grant" | **Limiters** – Abstract Available; Published Date: 20060101-20171231; English Language; Human. **Search modes** – Find any of my search terms | 26,104 |
| **S3** | S1 OR S2 | **Limiters** – Abstract Available; Published Date: 20060101-20171231; English Language; Human. **Search modes** – Find any of my search terms | 29,094 |
| **S4** | (MH "Clinical Research") OR (MH "Health Services Research") OR (MH "Research, Medical") | **Limiters** – Abstract Available; Published Date: 20060101-20171231; English Language; Human. **Search modes** – Find any of my search terms | 9,079 |
| **S5** | “health research” | **Limiters** – Abstract Available; Published Date: 20060101-20171231; English Language; Human. **Search modes** – Find any of my search terms | 40,925 |
| **S6** | S4 OR S5 | **Limiters** – Abstract Available; Published Date: 20060101-20171231; English Language; Human. **Search modes** – Find any of my search terms | 48,958 |
| **S7** | (MH “Health Impact Assessment”) | **Limiters** – Abstract Available; Published Date: 20060101-20171231; English Language; Human. **Search modes** – Find any of my search terms | 342 |
| **S8** | "research impact" OR " research value" OR " research outcom*" OR "research contribution*" OR “economic impact” | **Limiters** – Abstract Available; Published Date: 20060101-20171231; English Language; Human. **Search modes** – Find any of my search terms | 92,204 |
| **S9** | S7 OR S8 | **Limiters** – Abstract Available; Published Date: 20060101-20171231; English Language; Human. **Search modes** – Find any of my search terms | 92,530 |
| **S10** | S3 AND S6 AND S9 | **Limiters** – Abstract Available; Published Date: 20060101-20171231; English Language; Human. **Search modes** – Find any of my search terms | 112 |

EBSCOhost CINAHL® SEARCH STRATEGY

| **#** | **Searches** | **Limiters/Expanders** | **Results** |
| --- | --- | --- | --- |
| **S1** | (MH “Budgets") OR (MH "Health Services Purchasing")OR (MH "Financial Support") OR (MH "Investments") OR (MH "Resource Allocation") OR (MH "Health Resource Allocation") OR (MH "Research Support") OR (MH "Training Support, Financial") | **Limiters** – Abstract Available; Published Date: 20060101-20171231; English Language; Human. **Search modes** – Find any of my search terms | 3,205 |
| **S2** | "scholarship" OR "funding" OR "grant" | **Limiters** – Abstract Available; Published Date: 20060101-20171231; English Language; Human. **Search modes** – Find any of my search terms | 172,191 |
| **S3** | S1 OR S2 | **Limiters** – Abstract Available; Published Date: 20060101-20171231; English Language; Human. **Search modes** – Find any of my search terms | 174,108 |
| **S4** | (MH "Clinical Research") OR (MH "Health Services Research") OR (MH "Research, Medical") | **Limiters** – Abstract Available; Published Date: 20060101-20171231; English Language; Human. **Search modes** – Find any of my search terms | 11,607 |
| **S5** | “health research” | **Limiters** – Abstract Available; Published Date: 20060101-20171231; English Language; Human. **Search modes** – Find any of my search terms | 1,843 |
| **S6** | S4 OR S5 | **Limiters** – Abstract Available; Published Date: 20060101-20171231; English Language; Human. **Search modes** – Find any of my search terms | 7,180 |
| **S7** | (MH “Health Impact Assessment”) | **Limiters** – Abstract Available; Published Date: 20060101-20171231; English Language; Human. **Search modes** – Find any of my search terms | 78 |
| **S8** | "research impact" OR " research value" OR " research outcom*" OR "research contribution*" OR “economic impact” | **Limiters** – Abstract Available; Published Date: 20060101-20171231; English Language; Human. **Search modes** – Find any of my search terms | 14,961 |
| **S9** | S7 OR S8 | **Limiters** – Abstract Available; Published Date: 20060101-20171231; English Language; Human. **Search modes** – Find any of my search terms | 15,031 |
| **S10** | S3 AND S6 AND S9 | **Limiters** – Abstract Available; Published Date: 20060101-20171231; English Language; Human. **Search modes** – Find any of my search terms | 94 |
