# Supplementary material for: The impact generated by publicly and charity-funded research in the United Kingdom: a systematic literature review
Source: Health Res Policy Syst. 2019 Feb 28;17:22. doi: 10.1186/s12961-019-0425-2 (PMC6394081; doi:10.1186/s12961-019-0425-2)
Supplement: Supplementary file 3 — Data extraction table. (DOCX 29 kb) [file 12961_2019_425_MOESM3_ESM.docx]

**Additional file 3**

| **Author/Year** | **Aims** | **Programme(s) assessed** | **Methods** | **Design** | **Main Findings** |
| --- | --- | --- | --- | --- | --- |
| Bunn et al, 2015 | To identify the impacts and likely impacts on health care, patient outcomes and value for money of Cochrane Reviews published by 20 NIHR-funded CRGs during the years 2007–11. | 20 NIHR funded Cochrane Review Groups (CRG) | Mixed-methods | - Questionnaire survey of editorial boards of CRGs - Documentary review (hand search of annual CRG reports) - Bibliometric analysis of existing sources - Qualitative interviews with guideline developers from NICE, SIGN and WHO | Knowledge: A total of 1,502 (out of a total 3,187) new and updated reviews were produced by the 20 NIHR-funded CRGs between 2007-2011.  Future research: questionnaire responses provided 40 examples where they felt reviews influences primary research and 13 that had been cited in a protocol.  Policy: 483 systematic reviews cited in 247 sets of guidance: 62 international, 175 national and 10 local.  Health sector benefits: Review authors and CRGs provided some examples of impact on practice or services, for example, safer use of medication, the identification of new effective drugs or treatments. There are potential savings through the reduction in the use of unproven or unnecessary procedures. |
| Glover et al., 2014 | To estimate the economic returns from UK public and charitable funded cancer-related research that arise from the net value of the improved health outcomes | The leading funders of cancer research in the UK were identified by examining the National Cancer Research Institute (NCRI) Cancer Research Database | Quantitative | - Econometric analysis, using time-series of funding and health benefits to calculate the internal rate of return (IIR) | Economic impact: In 2011/12 prices, the net monetary benefit (NMB) of the 5.9 million QALYs gained from the prioritised interventions from 1991 to 2010 was £124 billion. Calculation of the IRR incorporated an estimated elapsed time of 15 years. The paper related 17% of the annual NMB estimated to be attributable to UK research (for each of the 20 years 1991 to 2010) to 20 years of research investment 15 years earlier (that is, for 1976 to 1995). This produced a best-estimate IRR of 10%.  Health gains: 5.9 million QALYs gained from the prioritised interventions from 1991 to 2010. |
| Guthrie et al., 2015 | To review the impact of NIHR HTA programme from 2003 to 2013, considering the following research impacts: academic, clinical practice, health and economic impacts. | NIHR’s Health Technology Assessment programme | Mixed methods | - Qualitative interviews with academics, policy-makers and the HTA programme - Bibliometric analysis - Quantitative survey of all HTA grant holders - Case studies | Knowledge: On average, work funded by the HTA is cited more than twice as frequently as would be expected on average.  Future research/research use: half of the studies were extended, in several cases new studies were shaped up, all had capacity building for their researchers.  Policy: 15% of the overall portfolio of studies reported having some impact on policy: Influenced training of practitioners, membership in a committee, citation in clinical reviews and policy documents, rapid advice and giving evidence to the government. Of these projects 60% were within the UK. Seven studies influences NICE guidelines and two the NSC pilot.  Health sector benefits: The primary root to impact is through guidelines (mainly NICE). Three of the cases studies reviewed showed benefits to patients in trauma, respiratory failure and stroke. Three studies showed impact on patient choice, two cases (limited) impact on potential cost savings, two cases showed current practice is appropriate.  Economic impact: little overlap between HTA and industry. Half of the studies showed impact on industry, potential impact through increased sales. |
| Guthrie et al., 2016 | To conduct an economic analysis of the impact of the HTA Programme | NIHR’s Health Technology Assessment programme | Mixed methods | - Analysis of the potential economic benefits of a sample of HTA funded studies and comparison to programme costs - Analysis of a set of short case studies exploring the impacts of the HTA Programme on policy and practice | Economic benefit: If 12 per cent of the potential net benefit of implementing the findings of this sample of 10 studies for one year was realised, it would cover the cost of the HTA Programme from 1993 to 2012.  Policy development: Three studies had a clear impact on policy through citation on guidance, with another expected to be included in guidance that is forthcoming.  Health sector benefits: Three studies showed a clear impact on practice. A further three cases showed some evidence of changes in practice but attributing that to the specific study is more challenging, though it is likely the study played a role. |
| Hanney et al., 2007 | To assess the impact of the first 10 years of the NHS HTA programme from its inception in 1993 to June 2003 and identify the factors that seem to be associated with HTA research making an impact. | NIHR’s HTA | Mixed methods | - A literature review of research programmes the work of the NCCHTA was reviewed - Survey of lead researchers - Analysis of 16 detailed case studies | Knowledge: Mean publications per project were 2.93 (1.98 excluding the monographs), above the level reported for other programmes.  Future research: The case studies confirmed the questionnaire responses but also showed how some projects led to further research and research capacity  Policy: The Technology Assessment Reports (TARs) for the National Institute for Health and Clinical Excellence (NICE) had the clearest impact on policy in the form of NICE guidance. Other bodies were the projects had impact included the National Screening Committee, the National Service Frameworks, professional bodies or the Department of Health. |
| Hanney et al., 2013 | This paper aims to describe the various impacts identified from a range of Asthma UK research. | Asthma UK | Mixed Methods | - Questionnaire survey - Qualitative interviews - Case studies | Knowledge: The 90 projects reported an average of four-peer reviewed journal articles per project. Four did not produce any.  Future research and career development: 64% reported career development opportunities as a result. 99 follow-up projects were developed, that received almost £25 million in funding from funders other than Asthma UK.  Policy and product development: Just 13% respondents claimed to have made an impact on policy, and 17% expected to do so in the future. In addition, 17% respondents claimed to have made an impact on product development already, and 31% expected to do so in the future.  Health gains: Only a small minority (10%) claim to have already made an impact in any of the various forms this could take, with 6% believing they had made an impact specifically to health.  Economic benefits: Limited evidence. Two spin-out companies were developed. |
| Lichten et al, 2017 | To evaluate the impact of the Oxford Biomedical Research Centre (Oxford BRC), a university-hospital partnership, on the effectiveness and efficiency of healthcare in local hospitals. | Oxford BRC (NIHR funded) | Qualitative | - Documentation review from Oxford BRC - Interviews with 17 leaders - Interviews with 19 senior clinicians | Health sector benefits: The research leaders identified a wide range of beneficial impacts that they expected might be felt at local hospitals as a result of their research activity.  The senior clinicians responsible for patient care at those hospitals presented a more mixed picture, identifying many positive impacts, but also a smaller number of negative impacts, from research activity, including that of the Oxford BRC. |
| Morgan Jones et al 2016 | To identify and synthesise evidence of NHIR funded research which has generated benefits to and wider impacts on the health research landscape. | NIHR | Synthesis report | - Analysis of 100 NIHR funded studies | Knowledge: Managing a shared knowledge resource: the Journals Library and BioResource.  Future Research/ Research Use: 10 cases showing collaboration with charities and the third sector; 10 cases showing patient involvement in research; 10 cases showing impact on training and developing workforce in the NHS and academia.  Policy and product development: 10 cases showing global impact; 10 cases showing investment across the nation.  Health sector benefits: 10 cases showing breakthroughs to patients; 10 cases improving the nation’s health and care system, 10 cases on public health delivery  Broader economic benefit: 10 cases creating opportunities for economic and social returns |
| McCrae et al, 2012 | To examine research network support to a multi-centre RCT of antidepressants in people with depression superimposed on dementia | The Mental Health Research Network (MHRN) and the Dementia and Neurodegenerative Diseases Research Network  (DeNDRoN).  Both networks are funded by the NIHR. | Qualitative | - Semi-structured interviews with PIs, research workers and research network officers | Research benefits: The MHRN helped gaining local ethics committee and NHS trust approvals, which can be a time-consuming process.  Clinical study officers boosted a recruitment campaign and contributed to the monitoring and assessment of participants.  Limitations: there were potential problems of duplication or unclear roles and responsibilities. There was also a degree of unrealistic expectation from principal investigators. The networks added to the bureaucratic burden. |
| Peckham et al., 2008 | To assess the impact of the first five years of the SDO Programme (2001–2006) | NHS Service Delivery and Organisation  Research and Development Programme (SDO) | Mixed methods | - Documentary and literature review - Bibliometric analysis - Qualitative analysis for 11 of those projects | Knowledge: of the 23 research projects, a total of 39 papers had been published in peer-reviewed journals by early 2006, equivalent to 1.7 articles per project. There were 95 national and international conference presentations. Each of the 23 research projects produced an average of 6.7 citations.  Future Research/ Research Use: The projects demonstrate contributions to building the capacity of the workforce, as there are many examples from the bibliographic analysis and the case studies where the knowledge is used in teaching in universities. There is some evidence that the research is stimulating user involvement in research.  Policy and product development: The literature review did not identify any citations in the documents related to this policy, but evidence from interviewees indicates other, informal mechanisms in which the knowledge was transmitted, such as meetings with the Department of Health. This demonstrates that knowledge can be effectively transferred in different ways but these may be difficult to trace when building an understanding of knowledge flow and research output. It has been difficult to confirm the use of SDO-funded research by practitioners through case studies. The data on outputs show that there is potential for a wide range of practitioners to access the research  Health Sector Benefits: The case studies demonstrate a range of ways in which NHS managers and policy makers have used SDO-funded research to develop service delivery. |
| Sainty, 2013 | To explore the wider scope of the research impact of UKOTRF – funded studies. | United Kingdom Occupational Therapy Research Foundation | Qualitative | - Assessment forms sent to eleven UKOTRF grant holders. Eight forms were returned | Knowledge: UKOTRF grant holders are required to submit an article to BJOT and an abstract for a COT Annual Conference. At the time of the study, one submission had been published, six participants had made submissions, and one submission was outstanding. Three participants had articles published in other peer-reviewed journals and one participant reported on co-authorship of a Cochrane review related to the research topic. Seven participants had made a submission, the eighth having presented in a UKOTRF session at conference.  Clinical applications: Three participants reported on how their project’s specific screening or assessment measures, mapping, or design tools had been demonstrated as valid or useful in clinical applications. No details provided.  Policy relevance: Strategic or policy perspective of impact was referred to by four participants. No details provided.  Research use: Some reference was made to educational activities, promotions and further research use. No details provided.  Economic benefits: The area of economic benefit impact was referred to by two participants, though the evidence was not very clear. |
| Sullivan et al., 2011 | To understand how cancer research actually maps to major centres and how this relates more broadly to policy-making both within and outside the domain of cancer. It compares pre- to post- NCRI periods. | The study compares the period before and after the launch of the National Cancer Research Institute (NCRI). | Quantitative | - Bibliographic analysis | Knowledge: UK centres published just over one eighth of the total UKCC in 1995 but almost a quarter by 2004.  Research use: There has been an increase in UKCC collaborations with European investigators (5–28% of all their outputs) and with USA (6% and 21%).  Policy development: There has been an increase in the number of citations on clinical guidelines and the press (BBC). |
| Sussex et al., 2016 | To estimate the magnitude of the effect of government and charity biomedical and health research expenditure in the UK, separately and in total, on subsequent private pharmaceutical sector R&D expenditure in the UK. | R&D expenditure from the MRC, the DoH and UK’s funding councils. | Quantitative | - Econometric analysis using data from biomedical and health R&D expenditure in the UK for ten disease areas (including ‘other’) for the government, charity and private sectors | Broad economic benefit: A 1 % increase/fall in public & charity biomedical and health research spend in the UK eventually is associated with a 0.81 % increase/fall in pharmaceutical industry R&D in the UK.  Overall, every additional £1 of public research expenditure is associated with an additional £0.83– £1.07 of private sector R&D spend in the UK; 44 % of that additional private sector expenditure occurs within 1 year, with the remainder accumulating over decades. This spillover effect implies a real annual rate of return (in terms of economic impact) to public biomedical and health research in the UK of 15– 18 %. When combined with previous estimates of the health gain that results from public medical research in cancer and cardiovascular disease, the total rate of return would be around 24– 28 %. |
